# *Polytraits*: A database on biological traits of marine polychaetes

**DOI:** 10.3897/BDJ.2.e1024

**Published:** 2014-01-17

**Authors:** Sarah Faulwetter, Vasiliki Markantonatou, Christina Pavloudi, Nafsika Papageorgiou, Kleoniki Keklikoglou, Eva Chatzinikolaou, Evangelos Pafilis, Georgios Chatzigeorgiou, Katerina Vasileiadou, Thanos Dailianis, Lucia Fanini, Panayota Koulouri, Christos Arvanitidis

**Affiliations:** † National and Kapodestrian University of Athens, Athens, Greece; ‡ Hellenic Centre for Marine Research, Heraklion, Crete, Greece; § Hellenic Centre for Marine Research, Heraklion, Greece; | Università Politecnica delle Marche, Ancona, Italy; ¶ Department of Biology, Faculty of Sciences, University of Ghent, 9000 Gent, Belgium, Department of Microbial Ecophysiology, Faculty of Biology, University of Bremen, 28359, Bremen, Germany; # Department of Biology, University of Patras, Rio, Patras, Greece

**Keywords:** Polychaeta, biological traits, functional traits, morphology, reproduction, behaviour, larvae, life cycle, life history, database, literature

## Abstract

The study of ecosystem functioning – the role which organisms play in an ecosystem – is becoming increasingly important in marine ecological research. The functional structure of a community can be represented by a set of functional traits assigned to behavioural, reproductive and morphological characteristics. The collection of these traits from the literature is however a laborious and time-consuming process, and gaps of knowledge and restricted availability of literature are a common problem. Trait data are not yet readily being shared by research communities, and even if they are, a lack of trait data repositories and standards for data formats leads to the publication of trait information in forms which cannot be processed by computers. This paper describes *Polytraits* (http://polytraits.lifewatchgreece.eu), a database on biological traits of marine polychaetes (bristle worms, Polychaeta: Annelida). At present, the database contains almost 20,000 records on morphological, behavioural and reproductive characteristics of more than 1,000 marine polychaete species, all referenced by literature sources. All data can be freely accessed through the project website in different ways and formats, both human-readable and machine-readable, and have been submitted to the Encyclopedia of Life for archival and integration with trait information from other sources.

## Introduction

Benthic organisms participate in a number of ecosystem-level processes, often described as “ecosystem functions”, which refer to any transformation process that occurs in an ecosystem (Cooper et al. 2008). The functional structure of a community can be represented by a set of functional traits assigned to behavioural, reproductive and morphological characteristics displayed by the observed species (Paganelli et al. 2012). Traits that affect resource use, feeding interactions, or habitat structure / availability are regarded as fundamentally important for ecosystem functioning (Bremner 2008), and the extent to which a species loss can threaten basic ecosystem processes depends on the functional richness (i.e., the number of functional groups) and evenness (i.e., the distribution of species across functional groups) in an ecosystem (Mouillot et al. 2005). Functional diversity is an important community property that can estimate the role organisms have in the ecosystem and can help to understand how the community reacts to environmental changes (Schleuter et al. 2010).

One approach to assess ecosystem functioning is to analyse species' biological traits which serve as a proxy for the functional characteristics of an assemblage (Bremner et al. 2003). This approach requires the selection of suitable traits that describe certain ecosystem processes and the assignment of species to these traits. However, this assignment is a laborious and time-consuming process that ideally requires collaborative work of a large team of people. Gaps of knowledge and information regarding species traits are a common problem (Tyler et al. 2012). Information may not be readily available since very often the relevant ecological and behavioural species studies are included in legacy literature which can be only found as rare paper copies in libraries around the world, or the biology of the species might not be known at all. Filling knowledge gaps requires both collection of additional data and development of statistical techniques for estimating missing trait values (Tyler et al. 2012). Lack of knowledge for certain traits in the literature is often surpassed by deriving available information for closely related species or even for species of the same family, since phylogenetically related species might have evolved similar environmental and ecological adaptations, thus leading to functional similarity (Usseglio-Polatera et al. 2000). However, the validity of this practice remains to be tested (Bevilacqua et al. 2012) and more accurate information on the evolutionary relationships between species is required (Tyler et al. 2012).

The organisation of the collected information in traits databases is an additional challenge. In most cases, trait data are not published together with the relevant manuscripts, or they are published as supplementary annexes in a format which is not machine-readable. The trend to share functional trait data over the internet, especially for the marine environment, is only recent. Examples of databases that provide trait information for marine species are the Neogene Marine Biota of Tropical America (NMiTA) database, the Biological Traits Information Catalogue (BIOTIC) of the Marine Life Information Network and SeaLifeBase. However, despite an increasing availability of these data through web-based databases, most of the data are not provided in a format that can be processed by computers. An obstacle to this is the lack of standardised data formats for describing trait data and the absence of ontologies (standardised and well-specified vocabulary of concepts and their interrelationships) with which trait information can be described. These are necessary prerequisites to integrate trait data into the semantic web – which will make them fully exploitable by both humans and computers. Furthermore, no public repository for traits data has existed to encourage authors to publish their data and make them re-usable. One initiative to address these problems is the TraitBank initiative by the Encyclopedia of Life (EOL), which will serve as a provider for aggregated species trait data in a machine-readable format.

This paper describes *Polytraits*, a database on biological traits of polychaetes (bristle worms, Polychaeta: Annelida). The database contains almost 20,000 records on morphological, behavioural and reproductive characteristics of more than 1,000 polychaete species (currently only marine species are covered). All data can be freely accessed through the project website in different ways and formats, both human-readable and machine-readable. Furthermore, the data are available through EOL's TraitBank, seamlessly integrating the information with other relevant trait data.

## General description

### Purpose

The project was initially started as an in-house project of the Hellenic Centre for Marine Research, within the framework of the MSc thesis of V. Markantonatou. Traits were collected for an ecological study of polychaetes in Mediterranean lagoons. Since then, the database has been continuously expanded to serve the data needs for other analyses (e.g. as part of the PhD thesis of S. Faulwetter). The database and website are being maintained by the Institute for Marine Biology, Biotechnology and Aquaculture of the Hellenic Centre for Marine Research and will be supported by and constitute a part of the LifeWatch Greece infrastructure.

## Project description

### Title

*Polytraits*: A database of biological traits of polychaetes collected from the literature

### Personnel

Sarah Faulwetter (Concept development, database and web development, data collection and management, trait definitions), Christos Arvanitidis (Concept development, data collection, trait definitions), Vasiliki Markantonatou (Concept development, trait definitions, data collection), Nafsika Papageorgiou (trait definitions, data collection), Christina Pavloudi (trait definitions, data collection), Kleoniki Keklikoglou (data collection), Georgios Chatzigeorgiou (data collection), Katerina Vasileiadou (data collection), Evangelos Pafilis (data collection, web service development), Thanos Dailianis (data collection, website design), Lucia Fanini (data collection), Eva Chatzinikolaou (data collection), Panayota Koulouri (data collection)

### Design description

**Figure 1.**
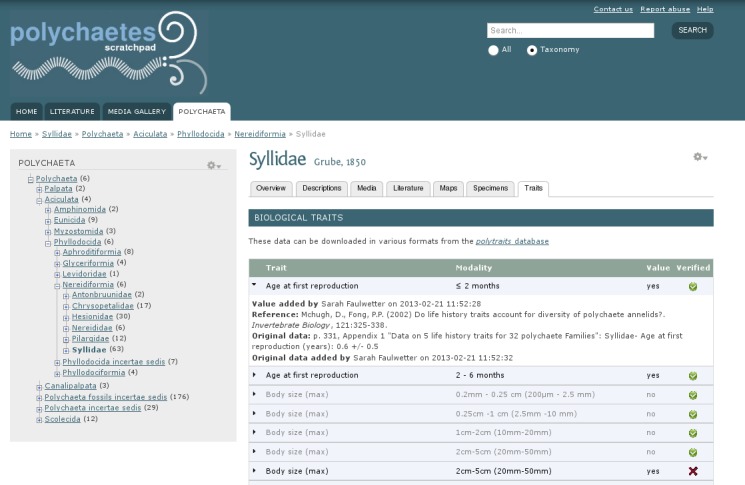
Screenshot of the Scratchpads interface to the *Polytraits* database.

To collect and disseminate biological trait data on polychaetes, a dedicated, web-based database has been created. It can be accessed at http://polytraits.lifewatchgreece.eu. The database features an entry interface (access only for registered users), and several options to access and export the data (see detailed documentation at the end of this manuscript). The data can be browsed furthermore a) through the Encyclopedia of Life and b) via a Scratchpads (http://www.scratchpads.eu) installation, dedicated to polychaete research (http://polychaetes.lifewatchgreece.eu). This taxon-centric virtual research environment (Smith et al. 2009) allows browsing the taxonomic classification and retrieving various kinds of relevant information for each taxon, among which are also the collected biological traits (Fig. 1).

The database contains 47 different traits describing the morphological, behavioural, reproductive and larval features as well as the environmental affinities of a taxon. Each trait has several sub-categories, so-called "modalities" (e.g. the trait "Mobility of the adult" contains the modalities "crawler", "burrower", "swimmer" and "non-motile/semi-motile"). In total, 252 modalities are covered. For a detailed list of all traits and modalities, including their definition, see section "Traits Coverage" below. The expression of a trait in a taxon is coded in the database by declaring each modality as "present" or "absent". This allows to capture both ambiguous information in the literature concerning the expression of a trait in a taxon, as well as the presence of multiple modalities in a taxon (e.g. some taxa can both be crawlers and swimmers).

Each assignment of a modality (and its presence/absence value) to a taxon is connected to a literature reference. This assignment is mandatory, no data can be entered without specifying the source of the data; however, to capture undocumented knowledge, the option "Expert's judgement" can be specified. The database contains its own literature management functions and stores references, the user can, during data entry, simply choose the desired reference from a list (or add it, if it is not yet present). It is possible to enter more than one reference per taxon-modality-value entry, thus corroborating the assignment. Additionally, most records are accompanied by the quotation of the exact literature passage which has led to the coding of the information. This serves two purposes: a) other researchers can re-use the data and code them differently if the modalities present in the database are not suitable for a specific analysis, and b) since the interpretation of text and the coding of the data is often subjective the original text can serve as a quality control mechanism. However, since this feature has only been introduced recently, some legacy data exist in the database which cite the reference but do not quote the exact text excerpt; these gaps are gradually being filled in.

## Sampling methods

### Study extent

The dataset includes biological traits (morphological, behavioural, reproductive and larval traits) of polychaetes. Since the data were initially collected with a specific research question and dataset in mind, a large number of the species in the database are typical inhabitants of European lagoons. However, this core set of species has been expanded over time and now comprises species from a large number of habitats and from world-wide locations. At present, the database contains only marine species, but freshwater and terrestrial polychaetes will be covered in the future.

### Sampling description

**Figure 2.**
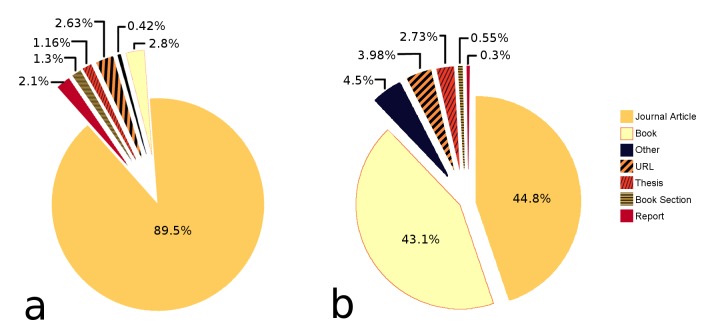
a) Different types of literature sources stored in the *Polytraits* database; b) Origin of the information in the *Polytraits* database. Most information is contained in books and review papers, whereas on average, a single research paper contains little information. Graph based on data in Suppl. material 4 and Suppl. material 5.

Data were collected from 950 different literature sources, the vast majority of which are scientific journals (Fig. 2a). However, on average most trait information is contained in books and review papers, whereas primary research papers often only contain information leading to the entry of one or very few records (Fig. 2b). Table 1 displays the references from which most of the trait data in the database originate. The system allows also to record experts' knowledge on taxa which is not documented in the literature; here the source of the data is specified as “Expert's judgement”. Literature references were not “sampled” exhaustively by systematically coding all contained trait information, but since the data collection was purpose-driven, only the information needed for the analysis at the time was coded. Table 1 therefore does not display the absolute trait information content for the listed references but for the data in the *Polytraits* database.

To enter the trait information, a dedicated web-based entry interface had been developed, assuring the integrity of the entered data and preventing information from being entered without specifying the literature source. The information found in the literature was assigned to pre-defined trait categories (“modalities”, see below under Trait coverage), specifying either “presence” or “absence” of a modality in a taxon (or both, if this information was found in the literature).

The system allows data entry at different taxonomic levels (from subspecies to family). Data was always assigned to the most specific taxon possible (e.g. the information “*Eteone longa* is a predator” was assigned to *Eteone longa*; the information “Some species of *Eteone* are predators” was assigned to *Eteone*, the information “All Sabellidae are filter feeders” was assigned to all taxa in the family Sabellidae). Information for taxa that are at present considered as subjective synonyms was entered for each respective taxon involved, and not for the taxon presently considered as "valid". This allows keeping information and taxonomy separate, and in case of a future resurrection of the synonymised taxon, the trait information does not have to be re-assigned. The structure of the database allows re-combining the information on synonyms during data export.

### Quality control

For each record, the relevant text excerpt from the source literature was recorded in addition to the citation (in the original language of the text, to avoid the introduction of translation errors). This short excerpt – usually no more than one or few short sentences – allows the reader to understand what led to the assignment of the taxon to a specific trait category. It also provides a means for quality control and allows other researchers to re-use the information in different contexts. This is especially helpful if the research question in mind requires different trait categories from those that have been chosen in this database.

Since the option to record the original text passage had been introduced at a later stage during the project, a certain percentage of the entries still lacks this information (see details below under "Taxonomic Coverage" and "Traits coverage", as well as Table 1) which is being added gradually to improve the quality of the data. The quality status is indicated in the Polychaetes Scratchpad trait section with either a green tick or a red cross in the rightmost column (Fig. 1).

### Step description

**Figure 3.**
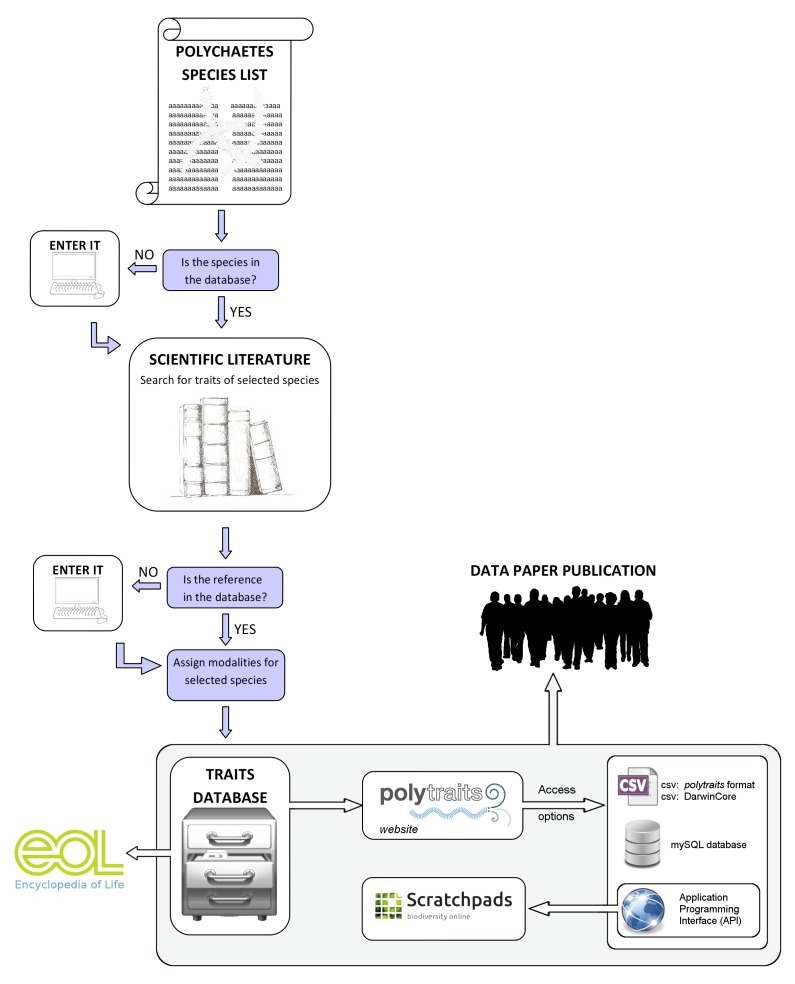
Flowchart of the data entry and publication process of the biological traits data in the *Polytraits* database. Starting with a species list for which data are to be collected, the respective taxa are entered in the database by the user. For each taxon, the required information is gathered from the literature. Data are always connected to their literature reference in the database and often the original text passage is also stored. Once in the database, the data can be accessed through an application programming interface (API), which also serves the Polychaetes Scratchpads, where data are displayed and can be browsed through a biological classification. Furthermore, data can be downloaded for offline use in various formats, and finally they can be browsed through the web portal of the Encyclopedia of Life.

The data collection for the *Polytraits* database has mainly been purpose-driven, meaning that information has been collected for a specific set of taxa for which an analysis was then performed. However, if no information on a certain trait in a taxon could be found in the literature, information for synonyms, congeners and confamiliars was likewise collected (from which data for the actual taxon in question can in some cases be derived). The data entry interface allows the registered user to enter new taxa and references. When a new taxon is entered, the taxonomic classification is automatically retrieved from the World Register of Marine Species and the taxon integrated into the local classification. All available information can be accessed and downloaded by the public through the *Polytraits* website, the Encyclopedia of Life, the Polychaetes Scratchpad or programmatically through a web service (see below under "Data resources" for further specifications of all access options). A schema of the data flow can be found in Fig. 3.

## Geographic coverage

### Description

A large number of taxa in the traits database are distributed in European waters. However, the database is constantly being expanded and by no means geographically restricted, so species from locations all over the world are also present in the database, albeit in lower numbers.

## Taxonomic coverage

### Description

**Figure 4.**
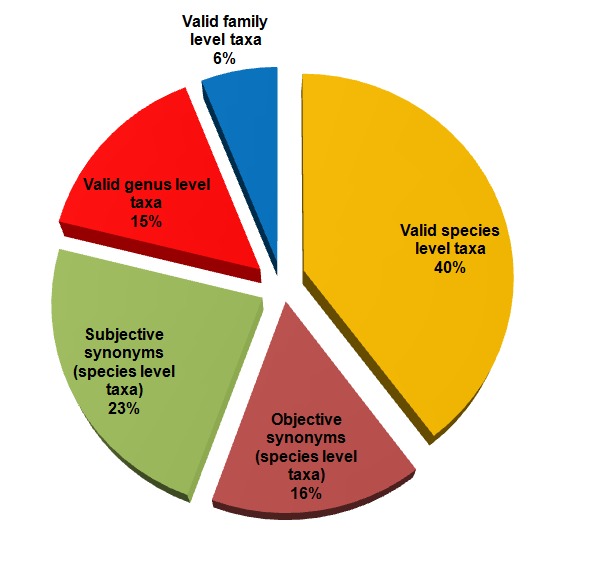
Taxonomic rank and status of taxa in the *Polytraits* database. Graph based on data in Suppl. material 6.

At present, the database contains 19,632 taxon-modality-value entries for 1,133 species-level, genus-level and family-level taxa of polychaetes. Currently only marine taxa are covered, but future expansions of the database will cover terrestrial and freshwater species as well. No data have been collected for taxa higher than the rank of family. Different representations of a taxonomic name can be stored in the database (e.g. objective synonyms, misspellings); the total number of taxonomic names for these taxa accounts to 1,373 (Fig. 4). A full list of all taxa in the *Polytraits* database for which trait information is available can be found on the website, including an overview of the amount of information available for each taxon. Several taxon-modality-value entries are supported by more than one literature source, so the total number of taxon-modality-value-reference entries amounts to 25,042. Updates to these numbers can be found on the *Polytraits* website.

**Figure 5.**
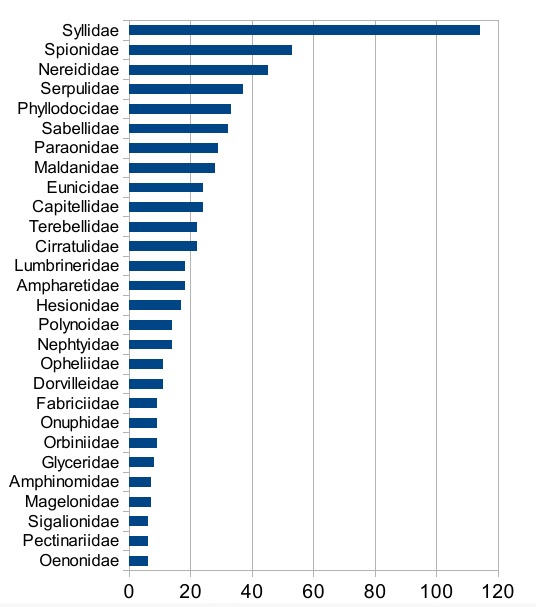
Number of valid species contained in the *Polytraits* database, per family. Graph based on data in Suppl. material 7.

**Figure 6.**
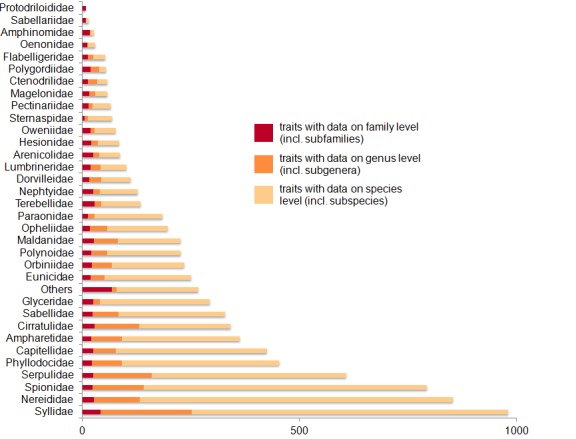
Number of taxon-modality-value records on family, genus and species level, per family. Graph based on data in Suppl. material 8.

Syllidae are the most species-rich family in the *Polytraits* database, followed by Spionidae and Nereididae (Fig. 5). Fig. 6 gives an overview of the granularity of the traits information available for each family: most information has been recorded at species level, though the percentage of information recorded for higher taxa (genus, family) is higher in some families with a low number of taxa.

The families with the largest number of species in the database contain also the largest amount of information on their biological traits. Table 2 gives an overview of the number of records per family (for all taxa within that family). The number of records for which the original text excerpt is not recorded varies among families. This is a result of the way information was recorded: during data collections in the beginning of the project the option to record this data was not implemented and this information was only gradually added. Taxa present in datasets for which traits data was collected recently have a higher percentage of recorded text excerpts.

### Taxa included

**Table:** 

Rank	Scientific Name	Common Name
kingdom	Animalia	Animals
phylum	Annelida	Segmented worms
class	Polychaeta	Bristle worms

## Traits coverage

The database contains 47 traits which are subdivided into 252 sub-categories (called "modalities"). They cover mainly reproductive and behavioural traits of both adult and larval stages, as well as information on environmental preferences and a few morphological traits.

Traits were chosen, defined and amended according to the needs of each analysis for which data was collected, thus they are a compilation of various sources. As a consequence several traits currently included have been recognised as inadequate to reflect polychaetes life histories and are likely to be changed (e.g. "Migration of adults", "Sociability", partly overlapping modalities of “Habitat” and “Physiographic feature”). Furthermore, the initial focus on European marine species has resulted in certain traits being defined appropriately for these taxa, but requiring an expansion of modalities if terrestrial and freshwater species, as well as species from other regions are included (e.g. expansion / refinement of salinity ranges, additional habitat terms). Future versions of this database will include a revised set of traits and the already existing data, but old versions will be available for download and changes to traits and their definitions will be properly documented.

No single suitable ontology (standardised vocabulary of concepts) exists to describe marine invertebrate traits. A sound definition of the concepts employed in the database and the identification of these concepts through Unique Resource Identifiers (URIs) allows the integration of the data into other trait data collections (such as the Encyclopedia of Life's TraitBank), therefore traits and modalities have been mapped, where possible, to existing ontology terms (e.g. the Environment Ontology or the Animal Natural History and Life History ontology).

Several traits and modalities are identical to those used by the BIOTIC database (MarLIN 2006), one of the most comprehensive databases on biological traits of marine organisms, to provide consistency across definitions and to ensure that data can be integrated more easily in the future. However, there is always a compromise required between trying to achieve comparability of the data and defining concepts as adequately as possible for a specific group of species, region and purpose. The approach used in this database is a mixed model – employing existing ontology concepts where possible, but defining others spedifically for the scope of this database. Future developments of the community concerning traits standards will therefore require continuous revisions of the traits and modalities used in the *Polytraits* database.

A full list of the traits, modalities and their definitions is given in Table 3. This table is a compact version of a more extensive documentation of the traits and modalities which can be found on the *Polytraits* website, where additional information and references for each trait and modality are provided, as well as ontology mappings and identifiers for each trait and modality in form of a Unique Resource Identifier (URI).

### Data coverage of traits

**Figure 7.**
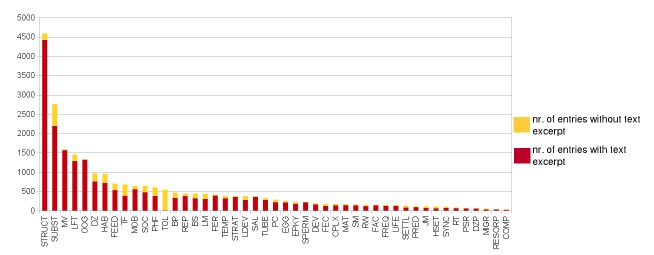
Traits coverage with the total number of entries and the number of entries with and without text excerpt. Graph based on data in Suppl. material 9. Abbreviations of traits as follows: **BP** = Parental care / Brood protection; **BS**= Body size (max); **COMP** = Intra- and Interspecies competition; **CPLX** = Complex species; **DEV** = Developmental mechanism; **DZ** = Depth zonation (benthos); **DZP** = Depth zonation (pelagic); **EGG** = Egg size; **EPKY** = Epitoky; **FAC** = Factors triggering reproduction; **FEC** = Fecundity; **FEED** = Feeding type; **FER** = Fertilization type; **FREQ** = Spawning frequency of a population; **HAB** = Habitat type; **HSET** = Habitat type of settlement/early development; **JMOB** = Juvenile mobility; **LDEV** = Larval development; **LFT** = Larval feeding type; **LIFE** = Lifespan; **LM** = Larval mode of development; **MAT** = Age at first reproduction; **MIGR** = Migrations of adult; **MOB** = Mobility of adult; **MV** = Metamorphosis volume; **OOG** = Pattern of oogenesis; **PC** = Location of parental care; **PHF** = Physiographic feature; **PRED** = Predated by; **PSR** = Population sex ratio; **REP** = Mode of reproduction; **RESORP** = Resorption of eggs; **RT** = Reproduction temperature; **RW** = Ecosystem engineering; **SAL** = Survival salinity; **SETTL** = Substrate type of settlement; **SM** = Sexual metamorphosis; **SOC** = Sociability; **SPERM** = Sperm type; **STRAT** = Reproduction strategy of the individual; **STRUCT** = Feeding structure; **SUBST** = Substrate type; **SYNC** = Synchronization of spawning; **TEMP** = Survival temperature; **TF** = Typically feeds on; **TOL** = Tolerance (AMBI index); **TUBE** = Tube / burrow material.

The amount of data available for the different traits varies greatly. The trait with the most entries is "Feeding structure", a morphological trait which is almost completely consistent within each family and known for all polychaete families. Information related to environmental preferences as well as mobility and feeding is likewise available for a large number of taxa, whereas many reproductive and larval traits are unknown for the majority of polychaetes (Fig. 7). Most of the information is furthermore accompanied by a quotation of the original text passage which helps interpreting the assignment of the trait to the taxon and provides a means of quality control Fig. 7. A certain percentage of the missing text excerpts is however attributed to the fact that for a trait assigned according to an expert's judgement, no text excerpt can be recorded.

**Figure 8.**
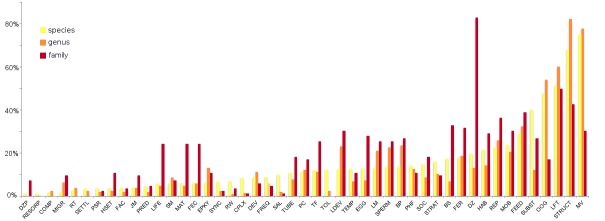
Percentage of number of species, genus and families for which information is available, per trait. Graph based on data in Suppl. material 10. Abbreviations of traits as in Fig. 7.

Fig. 8 provides a more detailed overview of the amount of information available per taxonomic rank for each trait. For several traits, information is mainly available for taxonomic ranks higher than species (e.g. information about depth distribution is known for most families, but less so for individual species).

## Temporal coverage

### Notes

**Figure 9.**
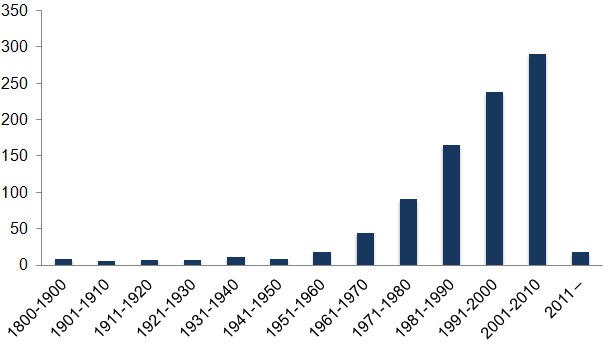
Temporal distribution (publication year) of references used to collect polychaete trait information. URLs and other references without a publication year are excluded from the graph. Graph based on data in Suppl. material 11.

To collect information on biological traits of polychaetes, literature from the past two centuries has been employed. However, the majority of the information was collected from literature published during the last decades Fig. 9.

## Usage rights

### Use license

Open Data Commons Attribution License

### IP rights notes

All data in the database can be freely used. Please cite this publication or the resource when using a large part of the data in your analyses. If individual records from the *Polytraits* database are displayed on a third-party website, it is recommended to cite also the record-level creator (see http://polytraits.lifewatchgreece.eu/download for more information), simply because often this information helps in assessing the quality of an entry (e.g. when "Expert's judgement" is given as a source for the information).

## Data resources

### Data package title

Polytraits database

### Resource link

http://polytraits.lifewatchgreece.eu

### Number of data sets

1

### Data set 1.

#### Data set name

Polytraits database

#### Data format

Darwin Core, csv, mySQL

#### Number of columns

10

#### Character set

UTF-8

#### Download URL

http://polytraits.lifewatchgreece.eu/download

#### Description

The data in the *Polytraits* database can be accessed in five different ways:

1. Browsing the data on a taxon-by-taxon basis through the Polychaetes Scratchpads (tab "Polychaetes" – tab "Traits").
2. Browsing the data through the Encyclopedia of Life (EOL).
3. Downloading the data as a comma separated value (*.csv) file from the *Polytraits* website. The download can be customised by submitting a list of taxa for which trait information should be retrieved or by limiting the output to specific traits. As an output format, either a *Polytraits*-specific format or DarwinCore can be chosen. Note that due to the restrictions of DarwinCore for trait data no information on the absence of a modality in a taxon could be included, this file therefore contains fewer records than the native export. A description of the returned fields for the *Polytraits*-specific format are documented in Table 4, those for the DarwinCore format are described below.
4. Downloading the full database as a MySQL script, automatically created at monthly intervals. A full documentation of all database tables and fields, including an Entity Relationship Diagram is provided in Suppl. material 1. The script can be imported into any local MySQL database and will automatically create all relevant tables and their data.
5. Accessing the data programmatically via a web service (REST API, application programming interface). This approach addresses a more technically oriented audience. The API can be accessed as follows: **http://polytraits.lifewatchgreece.eu/{method}/{query}/{format}/?{other parameter key value pairs}**. Two methods are currently available (documented in Table 5 and Table 6; an exemplary client implementing both methods is provided both as a PHP and a perl script in Suppl. material 2):
  1. ***taxon***: searches for a taxon name and returns taxon identifier(s);
  2. ***traits***: retrieves traits information for one or more taxon identifiers.

Whereas data downloaded as csv files or accessed through the API always reflect the latest changes in the database, the MySQL export is provided as monthly snapshots. The data available through EOL are a one-time export and reflect the database contents as of November 6th, 2013.

**Data set 1.** 

Column label	Column description
scientificName	The taxon for which the information was recorded.
scientificNameAuthorship	The author and year of the taxon for which the information was recorded.
acceptedNameUsage	Currently accepted name and authorship of the scientificName (as stored in the Polytraits database – information might not be up to date with the latest taxonomic literature in some cases).
taxonomicStatus	The status of the use of the scientificName (e.g. objective synonym, subjective synonym) as stored in the Polytraits database. Empty if scientificName is the currently accepted name.
MeasurementOrFact	Unique Resource Identifier pointing to the definition of a biological trait.
measurementValue	Unique Resource Identifier pointing to the definition of a modality (trait category) which is expressed by the current taxon.
dcterms:bibliographicCitation	Full literature reference (including Digital Object Identifier (DOI) where present) supporting the trait information for the current taxon.
measurementRemarks	A quotation of the original text passage containing the trait information for the current taxon.
measurementDeterminedBy	Person who entered the trait information for this taxon into the database.
measurementDeterminedDate	Date the trait information was entered into the database or last modified.

## Additional information

**Resource citation:** Polytraits Team (2013 –) *Polytraits*: A database on biological traits of polychaetes, 25034 data records, Contributors: Faulwetter S, Markantonatou V, Pavloudi C, Papageorgiou N, Keklikoglou K, Chatzinikolaou E, Pafilis E, Chatzigeorgiou G, Vasileiadou K, Dailianis T, Fanini L, Koulouri P, Arvanitidis C. Online at http://polytraits.lifewatchgreece.eu. Version 1.0, last updated on: 2013-11-14.

## Supplementary Material

Supplementary material 1 Database documentation Data type: pdf document
Brief description: Document describing all database tables that are made available to the user for download through the *Polytraits* website. Contains both a detailed description of all database fields as well as a diagram of the tables' relationships.
File: oo_5039.pdf Sarah Faulwetter

Supplementary material 2 Polytraits REST API Data type: php / perl script (zipped)
Brief description: Exemplary PHP and perl client demonstrating the two data retrieval methods currently implemented by the *Polytraits* database.
File: oo_4990.zip Sarah Faulwetter

Supplementary material 3 Full list of literature references Data type: csv file with literature references
Brief description: Full list of all literature references from which data for the *Polytraits* database were collected.
File: oo_4755.csv Sarah Faulwetter

Supplementary material 4 Literature sources used. Data type: Excel file with summarized data from database
Brief description: Types of literature sources used in the *Polytraits* database.
File: oo_5155.xls Sarah Faulwetter

Supplementary material 5 Origin of trait information Data type: Sarah Faulwetter
Brief description: Origin (literature type) of the traits information in the database.
File: oo_5157.xls Sarah Faulwetter

Supplementary material 6 Taxon ranks and status Data type: Excel file with summarized data from database
Brief description: Taxonomic rank and status of taxa in the *Polytraits* database.
File: oo_5156.xls Sarah Faulwetter

Supplementary material 7 Number of species Data type: Excel file with summarized data from database
Brief description: Number of valid species contained in the *Polytraits* database, per family.
File: oo_5158.xls Sarah Faulwetter

Supplementary material 8 Number of records Data type: Excel file with summarized data from database
Brief description: Number of taxon-modality-value records on family, genus and species level, per family.
File: oo_5159.xls Sarah Faulwetter

Supplementary material 9 Traits coverage Data type: Excel file with summarized data from database
Brief description: Traits coverage with the total number of entries and the number of entries with and without text excerpts.
File: oo_5547.xls Sarah Faulwetter

Supplementary material 10 Completeness of traits Data type: Excel file with summarized data from database
Brief description: Percentage of number of species, genus and families for which information is available, per trait.
File: oo_5161.xls Sarah Faulwetter

Supplementary material 11 Temporal distribution of literature Data type: Excel file with summarized data from database
Brief description: Temporal distribution (publication year) of references which were used in the *Polytraits* database.
File: oo_5162.xls Sarah Faulwetter

## Figures and Tables

**Table 1.** The most employed literature references (more than 100 taxon-modality records each) in the *Polytraits* database. The number of taxon-modality records supported by each reference is given, as well as the number of records for which the original text excerpt is quoted (see also section "Quality control"). A full list of all references used to collect data for the *Polytraits* database can be found in Suppl. material 3.

Reference	Nr. of records (total)	Nr. of records withtext excerpt	Nr. of records withouttext excerpt
Rouse and Pleijel (2001)	5466	5466	0
Rouse and Pleijel (2006)	2252	2252	0
Hartmann-Schröder (1996)	1756	1755	1
Almeida et al. (2003)	1540	1540	0
Expert's Judgement	1131	763	368
MarLIN (2006)	530	324	206
Eckelbarger (2005)	502	497	5
Borja et al. (2000)	491	1	490
Fauchald and Jumars (1979)	452	327	125
Arvanitidis (1994)	450	220	230
San Martín (2003)	410	388	22
Wilson (1991)	346	324	22
Read (2006)	214	214	0
Vieitez et al. (2004)	203	201	2
Carson and Hentschel (2006)	201	34	167
Blake and Arnofsky (1999)	181	120	61
Tzetlin and Purschke (2005)	178	140	38
Holthe (1986)	139	117	22
Fauvel (1927)	126	23	103
Wolff (1973)	124	124	0
McHugh and Fong 2002	114	82	32
Kupriyanova et al. 2001	111	59	52

**Table 2.** Number of taxon-modality-literature records per family (for all taxa in each family), as well as the number and percentage of these records for which the original text excerpt from the literature has also been recorded.

Family	Total nr. of records	Nr. of entries withtext excerpt	% of entries with text excerpt	% of entries without text excerpt
Nereididae	3168	3012	95.08	4.92
Syllidae	2860	2541	88.85	11.15
Spionidae	2190	1758	80.27	19.73
Serpulidae	1820	1529	84.01	15.99
Capitellidae	1469	1331	90.61	9.39
Phyllodocidae	1257	1189	94.59	5.41
Cirratulidae	1171	696	59.44	40.56
Ampharetidae	1103	987	89.48	10.52
Sabellidae	910	740	81.32	18.68
Glyceridae	818	740	90.46	9.54
Orbiniidae	710	532	74.93	25.07
Eunicidae	644	509	79.04	20.96
Polynoidae	615	562	91.38	8.62
Opheliidae	568	501	88.2	11.8
Paraonidae	561	453	80.75	19.25
Maldanidae	516	462	89.53	10.47
Terebellidae	500	259	51.8	48.2
Nephtyidae	480	422	87.92	12.08
Arenicolidae	455	121	26.59	73.41
Lumbrineridae	356	136	38.2	61.8
Oweniidae	339	152	44.84	55.16
Pectinariidae	283	257	90.81	9.19
Dorvilleidae	279	233	83.51	16.49
Hesionidae	279	176	63.08	36.92
Fabriciidae	278	234	84.17	15.83
Sternaspidae	197	191	96.95	3.05
Flabelligeridae	164	121	73.78	26.22
Magelonidae	153	63	41.18	58.82
Ctenodrilidae	152	113	74.34	25.66
Polygordiidae	130	76	58.46	41.54
Oenonidae	115	42	36.52	63.48
Trichobranchidae	71	58	81.69	18.31
Amphinomidae	60	55	91.67	8.33
Sabellariidae	33	31	93.94	6.06
Paralacydoniidae	32	32	100	0
Lacydoniidae	23	23	100	0
Chrysopetalidae	23	23	100	0
Protodriloididae	19	19	100	0
Pholoidae	18	18	100	0
Sphaerodoridae	16	16	100	0
Siboglinidae	10	10	100	0
Myzostomidae	9	9	100	0
Aberrantidae	8	8	100	0
Aphroditidae	8	7	87.5	12.5
Goniadidae	8	8	100	0
Onuphidae	8	8	100	0
Acrocirridae	7	7	100	0
Chaetopteridae	7	7	100	0
Pilargidae	7	7	100	0
Tomopteridae	7	7	100	0
Acoetidae	6	6	100	0
Aeolosomatidae	6	6	100	0
Alvinellidae	6	6	100	0
Fauveliopsidae	6	6	100	0
Nerillidae	6	6	100	0
Pisionidae	6	6	100	0
Poecilochaetidae	6	6	100	0
Parergodrilidae	6	6	100	0
Alciopidae	6	6	100	0
Cossuridae	5	5	100	0
Euphrosinidae	5	5	100	0
Scalibregmatidae	5	5	100	0
Sigalionidae	5	5	100	0
Nautiliniellidae	4	4	100	0
Questidae	4	4	100	0
Typhloscolecidae	4	4	100	0
Alciopidae	4	4	100	0
Eulepethidae	3	3	100	0
Histriobdellidae	3	3	100	0
Psammodrilidae	3	3	100	0
Spintheridae	3	3	100	0
Endomyzostomatidae	2	2	100	0
Lopadorhynchidae	2	2	100	0
Poeobiidae	2	2	100	0
Trochochaetidae	2	2	100	0
Apistobranchidae	1	1	100	0
Flotidae	1	1	100	0
Hartmaniellidae	1	1	100	0
Uncispionidae	1	1	100	0

**Table 3.** Definition of traits and their modalities in the *Polytraits* database. Please refer to the extended version of this table at http://polytraits.lifewatchgreece.eu/terms for additional descriptions and literature references, related terms and synonyms, as well as Unique Resource Identifiers (URIs) and ontology mappings for each trait and modality.

***Adult traits***	
**Body size (max)**	A measurement of the longest dimension of a body, typically between two distinct ends of the body. In polychaetes, this is the length from the head to the pygidium without appendages like antennae or cirri. In the *Polytraits* database there are 7 different classes (modalities) for this trait. For the coding of modalities, the maximum body size that is reported in the literature for a species is chosen.
Modalities:	
*< 2.5 mm*	Maximum body size up to 2.5 mm.
*2.5 mm – 10 mm*	Maximum body size from 2.5 to 10 mm.
*11 mm – 20 mm*	Maximum body size from 11 to 20 mm.
*21 mm – 50 mm*	Maximum body size from 21 to 50 mm.
*51 mm – 80 mm*	Maximum body size from 51 to 80 mm.
*81 mm – 100 mm*	Maximum body size from 81 to 100 mm.
*> 100 mm*	Maximum body size more than 100 mm.
**Complex species**	A group of species which satisfy the biological definition of species, that is, they are reproductively isolated from each other, but they are not morphologically distinguishable (or at least are not readily or reliably distinguishable on a morphological basis) (Mayr and Ashlock 1991).
Modalities:	
*yes*	Complex species reported in the literature.
*no*	No complex species reported in the literature.
**Depth zonation (benthos)**	The depth at which an organism occurs. Commonly defined based on ecological features of the zonation.
Modalities:	
*supralittoral zone*	The zone of the shore immediately above the highest water level and subjected to wetting by spray or wave splash (Lincoln et al. 1998).
*littoral zone*	The area of the foreshore and seabed that is exposed to the air at low tide and submerged at high tide, i.e., the area between tide marks.
*sublittoral zone*	The zone of the shore immediately below the lowest water level and the edge of the continental shelf (ca. 200 m).
*bathyal zone*	The steep descent zone from 200 m to 4000 m depth.
*abyssal zone*	The zone between 4000 – 6000 m depth (Lincoln et al. 1998).
*hadal zone*	The sea floor deeper than 6000 m, such as that of the oceanic trenches.
**Depth zonation (pelagic)**	The depth at which an organism occurs in the water column. Commonly defined based on ecological features of the zonation.
Modalities:	
*epipelagic zone*	The zone of an ocean from the surface to 200 m where photosynthesis can occur, due to the penetration of light.
*mesopelagic zone*	Water column from the upper aphotic zone (ca. 200 m) to a depth of ca. 1000 m (MarLIN 2013).
*bathypelagic zone*	Water column from ca. 1000 m to a depth of ca. 2500 m (MarLIN 2013).
*abyssopelagic zone*	The zone of the ocean below the bathypelagic zone, with its lowest boundary at about 6000 m.
*hadalpelagic zone*	The zone of an ocean in oceanic trenches, lying between 6000 m and 10000 m.
**Ecosystem engineering**	Organisms can be considered as ecosystem engineers when they directly or indirectly modulate the availability of resources to other species, by causing physical state changes in biotic or abiotic materials. In so doing they modify, maintain and/or create habitats (Jones et al. 1994).
Modalities:	
*yes*	“Umbrella term”. Used to capture information that a species is an ecosystem engineer, without specifying the type of engineering.
*no*	“Umbrella term”. Used to capture information that a species is not an ecosystem engineer.
*biodiffusor*	Biodiffusors include organisms with activities that usually result in a constant and random local sediment biomixing over short distances (Kristensen et al. 2012).
*upward conveyor*	Upward conveyors are vertically oriented species that typically feed head-down at depth in the sediment. Vertically oriented head-down feeders actively select and ingest particles at the deeper sediments and egest these non-locally as faeces in the sediment surface (Kristensen et al. 2012).
*downward conveyour*	Downward conveyors exhibit a feeding strategy opposite to that of upward conveyors. Vertically oriented head-up feeders actively select and ingest particles at the surface and egest these non-locally as faeces in deeper sediment strata (Kristensen et al. 2012).
*regenerator*	Regenerators are excavators that dig and continuously maintain burrows in the sediment and by doing so they mechanically transfer sediment from depth to the surface.
*blind-ended ventilation*	Ventilation occurs when animals flush their burrows with overlying water for respiratory and feeding purposes. Blind-ended ventilation occurs when I-shaped burrows are flushed uni- or bidirectionally depending on the permeability of the sediment (Kristensen et al. 2012).
*open-ended ventilation*	In open-ended ventilation the burrows are U-shaped and can be flushed easily from one end to the other (Kristensen et al. 2012).
*habitat-building (reef-forming)*	Species which create structures which in turn form new habitats for other species.
**Feeding structure**	The feeding structures of the polychaetes vary, reflecting the diversity of feeding types. There are two major anatomical/morphological features involved in the polychaetes feeding: the pharynx and the feeding structures of the prostomium (e.g. palps) (Rouse and Pleijel 2006).
Modalities:	
*simple axial pharynx*	A sac-like pharynx relying on fluid pressure from the coelom for eversion (Rouse and Pleijel 2006).
*ventral buccal organ (simple)*	A variable set of folds, musculature and glands, present on the ventral side of many polychaetes, is usually referred to as a ventral pharynx and is the most common form in Polychaeta (Rouse and Pleijel 2006).
*ventral muscularpharynx*	The ventral and lateral walls of the buccal region are muscular and the lining is sclerotized into a varying number of eversible jaw pieces. The jaws are separated into a pair of ventral mandibles and two or more pairs of lateral maxillae (Rouse and Pleijel 2006).
*muscular axialpharynx*	The pharynx has thickened, strongly muscular walls and can be retracted into a sheath. In other cases the pharynx is partially retracted and partially inverted. The mouth proper is located at the tip of the pharynx when fully everted (Rouse and Pleijel 2006).
*buccal organ absent or occluded*	The buccal cavity lacks obvious differentiation of the wall and it is not eversible. In some species, if the buccal cavity is present at all, it is only a transient larval structure and becomes completely occluded (Rouse and Pleijel 2006).
*accessory feeding structures*	Other structures as palps, tentacles or a radiolar crown ("grooved palps").
**Feeding type**	The common diet of an organism that includes the food items which are enzymatically and behaviourally capable of using.
Modalities:	
*predator*	An organism that feeds by preying on other organisms, killing them for food (MarLIN 2013).
*suspension feeder*	Any organism which feeds on particulate organic matter, including plankton, suspended in the water column (MarLIN 2013).
*non-selective deposit feeder*	An organism that feeds on mud or sand and may show a little discrimination in the size or type of particles eaten. The sediment is ingested and any digestible organic material is assimilated as it passes through the alimentary canal.
*selective deposit feeder*	Some deposit feeders do not ingest sediment haphazardly but use their palps or buccal organs to sort organic material from the sediment prior to ingestion. The method of sorting varies according to the types of palps present.
*deposit feeder (selective or non-selective)*	“Umbrella term”. Any organism which feeds on fragmented particulate organic matter from the substratum (MarLIN 2013). This modality should be filled in if nothing about the selectivity of the deposit feeding is known.
*omnivore*	Organisms which feed on a mixed diet including plant and animal material (MarLIN 2013).
*scavenger*	Any organism that actively feeds on dead animals.
*herbivore*	An animal that feeds on plants or algae, or parts of them.
**Habitat type**	The place in which an organism lives. It is defined for the marine environment according to geographical location, physiographic features as well as the physical and chemical environment (including salinity, wave exposure, strength of tidal streams, geology, biological zone, substratum, 'features' (e.g. crevices, overhangs, rockpools) and 'modifiers' (e.g. sand-scour, wave-surge, substratum mobility) (MarLIN 2013). The modalities of this trait might be expanded in the future and/or merged with the trait "Physiographic feature".
Modalities:	
*algae*	Macroalgae surfaces, such as *Laminaria* spp., or fucoids.
*biogenic reef*	Solid, massive structure which is created by accumulations of organisms, usually rising from the seabed, or at least clearly forming a substantial, discrete community or habitat which is very different from the surrounding seabed. The structure of the reef may be composed almost entirely of the reef building organism and its tubes or shells, or it may to some degree be composed of sediments, stones and shells bound together by the organisms (Holt et al. 1998).
*caves*	A hollow normally eroded in a cliff, with the penetration being greater than the width at the entrance (Sunamura 1992). Caves can also be formed by boulders (MarLIN 2013).
*crevices / fissures*	Crevices are narrow cracks in a hard substratum < 10 mm wide at its entrance, with the penetration being greater than the width at the entrance. Fissures are cracks in a hard substratum > 10 mm wide at its entrance, with the depth being greater than the width at the entrance (MarLIN 2013).
*maerl / coralligenous habitats*	A coralligenous habitat is defined by the presence of a bioherm of coralline algae grown at low irradiance levels and in relatively calm waters (Ballesteros 2006). Maerl denotes loose-lying, normally non-geniculate (i.e. not jointed), coralline red algae. Depending on the terminology used, maerl refers either to a class of rhodoliths, or may be considered distinct from rhodoliths in lacking a non-algal core. Maerl beds are composed of living or dead unattached corallines forming accumulations with or without terrigenous material (Birkett et al. 1998).
*other species*	Epibiont of other species.
*overhangs*	An overhanging part of a rock formation.
*rockpools*	A depression in the littoral zone of a rocky seashore, where, during low tide, seawater is left behind (MarLIN 2013).
*salt marsh*	A marsh whose water contains a considerable quantity of dissolved salts.
*seagrass*	Habitat associated with seagrass meadows communities. Seagrasses are flowering plants that are adapted to living fully submerged and rooted in estuarine and marine environments (MarLIN 2013).
*strandline*	A line on the shore composing debris deposited by a receding tide; commonly used to denote the line of debris at the level of extreme high water (MarLIN 2013).
*under boulders*	Under unattached rocks that can be very large (> 1024 mm), large (512 – 1024 mm) or small (256 – 512 mm) (MarLIN 2013).
*water column*	Pelagic habitat.
*soft sediments*	Deposits with a high water content (near or above the liquid limit), where the percolating skeleton is made of fine-grained soils (clay fraction above ~ 20%), with a high degree of saturation, and subjected to low effective confinement (Klein and Santamarina 2005).
**Intra- and interspecific competition**	The simultaneous demand by two or more organisms or populations or species for an essential common resource that is actually or potentially in limited supply or the detrimental interaction between two or more organisms or species seeking a common resource that is not limited (Eleftheriou 1997).
Modalities:	
*annelida (adults)*	Competition with other annelids that are in adult stage. The interaction can be between different organisms, populations or species.
*crustacea (adults)*	Competition with crustaceans that are in adult stage.
*annelida (larvae)*	Competition with other annelids that are in larval stage. The interaction can be between different organisms, populations or species.
*crustacea (larvae)*	Competition with crustaceans that are in larval stage.
*mollusca*	Competition with mollusks.
**Lifespan**	Maximum length of time that any particular organism can be expected to live.
Modalities:	
*≤ 1 year*	Life span shorter than a year.
*1 – 3 years*	Life span between 1 and 3 years.
*3 – 5 years*	Life span between 3 and 5 years.
*≥ 5 years*	Life span more than 5 years.
**Migrations of adult**	Movement of an organism or group from one habitat or location to another (MarLIN 2013). This trait is poorly defined for invertebrates. Within the context of this database it will likely be redefined or become obsolete.
Modalities:	
*yes*	“Umbrella term”. Used to capture information that a species in its adult stage is migratory.
*no*	“Umbrella term”. Used to capture information that a species in its adult stage is non-migratory and remains within the same area.
**Mobility of adult**	The capability of an organism to move spontaneously and freely (MarLIN 2013).
Modalities:	
*crawler*	An organism that moves along on the substratum via movements of its legs, appendages (e.g. parapodia and chaetae) or muscles (MarLIN 2013).
*burrower*	An organism that lives or moves in a burrow in soft sediments.
*swimmer*	An organism that swims through the water column via movements of its fins, legs or appendages, via undulatory movements of the body or via jet propulsion; includes pelagic phases during reproduction (swarming at the surface) (MarLIN 2013).
*non-motile / semi-motile*	Permanently attached to a substratum (non-motile) or capable of movement across (or through) it (semi-motile) (MarLIN 2013).
**Physiographic feature**	The general physical characteristics of the marine environment in which an organism lives (MarLIN 2013). The modalities of this trait might be expanded in the future and /or merged with the trait "Habitat".
Modalities:	
*open coast*	Any part of the coast not within a marine inlet, strait or lagoon, including offshore rocks and small islands. This includes MNCR types; Linear coast, Islands / Rocks and Semi-enclosed coast.
*offshore seabed*	Seabed beyond three miles (5 km) from the shore.
*strait*	Strait is a narrow channel of water that connects two larger bodies of water, and thus lies between two land masses.
*fjord*	Fjord is a long and narrow sea inlet with high steeply sloped walled sides. A fjord is a landform created during a period of glaciation. Includes also sea lochs.
*ria*	Ria is a submergent coastal landform where sea levels rise either in relation to the land or as a result of eustatic sea level change; where the global sea levels rise or isostatic sea level change; where the land sinks. When this happens valleys which were previously at sea level become submerged. Includes also voes.
*estuary*	A semi-enclosed coastal body of water with one or more rivers or streams flowing into it, and with a free connection to the open sea.
*enclosed coast / embayment*	An area of water bordered by land on three sides. Includes also harbours and marinas.
*lagoon*	Enclosed bodies of water separated or partially separated from the sea by shingle, sand or sometimes rock and with a restricted exchange of water with the sea, yielding varying salinity regimes.
*hydrothermal vents*	A marine hydrothermal vent is a marine benthic feature where heat generated due to tectonic activity, either at divergent plate boundaries or convergent ocean plates where back-arc spreading occurs, is released or 'vented' to the surface. The resultant high temperature water jets are laden with dissolved metals and minerals.
**Predated by**	Organism categories that feed by preying on the present species. The modalities of this trait might be expanded in the future.
Modalities:	
*annelids*	Prey for other annelids.
*crustaceans*	Prey for crustacean species.
*fish*	Prey for fish species.
*birds*	Prey for bird species.
*mollusks*	Prey for mollusks.
*echinoderms*	Prey for echinoderm species.
**Sociability**	“Umbrella term”. Used to capture information that an organism, population or species is living alone or interacting with others forming groups/communities or colonies (through asexual reproduction). This term and its modalities are poorly defined and will likely be re-defined within the context of this database.
Modalities:	
*algae*	Species that interact/live with algae.
*seagrasses*	Species that interact/live with seagrass meadows.
*annelids*	Species that interact/live with annelids.
*bacteria*	Species that interact/live with bacteria.
*crustaceans*	Species that interact/live with crustaceans.
*fish*	Species that interact/live with fish.
*mollusks*	Species that interact/live with mollusks.
*nematodes*	Species that interact/live with nematodes.
*echinoderms*	Species that interact/live with echinoderms.
*cnidarians*	Species that interact/live with cnidarians.
*poriferans*	Species that interact/live with poriferans.
*branchiostomids*	Species that interact/live with branchiostomids.
*bryozoans*	Species that interact/live with bryozoans.
*entoproctans*	Species that interact/live with entoproctans.
**Substrate type**	The surface on which an organism lives. The substrate may simply provide structural support, or may provide nutrients (MarLIN 2013).
Modalities:	
*bedrock*	Any stable hard substratum not separated into boulders or smaller sediment units.
*large to very large boulders*	Unattached rock, of large (512 – 1024 mm) or very large (> 1024 mm) size (MarLIN 2013).
*small boulders*	Unattached rock, of small (256 – 512 mm) size (MarLIN 2013).
*cobbles*	Sediment characterised by an average particle diameter between 64 and 256 mm.
*pebbles*	Sediment characterised by an average particle diameter between 4 and 64 mm.
*gravel*	An environmental material which is composed of pieces of rock that are at least two millimeters (2 mm) in its largest dimension and no more than 75 millimeters.
*sandy gravel*	50 – 80% gravel; 20 – 50% sand.
*muddy gravel*	50 – 80% gravel; 20 – 50% mud.
*muddy sandy gravel*	50 – 80% gravel; 20 – 50% mud and sand.
*coarse clean sand*	Sediment particles diameter between 0.5 – 4 mm; the sand fraction is > 80%.
*fine clean sand*	Sediment particles diameter between 0.063 – 0.5 mm; the sand fraction is > 80%.
*gravelly sand*	50 – 80% sand; 20 – 50% gravel.
*muddy gravelly sand*	50 – 80% sand; 20 – 50% mud and sand.
*muddy sand*	50 – 80% sand; 20 – 50% mud.
*sandy mud*	50 – 80% mud; 20 – 50% sand.
*sandy gravelly mud*	50 – 80% mud; 20 – 50% sand and gravel.
*gravelly mud*	50 – 80% mud; 20 – 50% gravel.
*mud*	Fine particles of silt and/or clay < 0.063 mm; the silt/clay fraction is > 80% (MarLIN 2013).
*silt*	Sediment characterised by an average particle diameter between 3.9 and 63 micrometers.
*clay*	Sediment characterised by an average particle diameter between 1 and 3.9 micrometers.
*mixed*	Mixtures of a variety of sediment types composed of pebble/gravel/sand/mud. This category includes muddy gravels, muddy sandy gravels, gravelly muds, and muddy gravelly sands.
*artificial*	E.g. wood, metal or concrete structures.
**Survival salinity**	The range of salinity in which an organism is capable to survive and grow (MarLIN 2013). The modalities of this trait will be refined in the future to capture also hypersaline and freshwater species.
Modalities:	
*full salinity*	The capability of an organism to live in environments of average marine water salinity (30 – 40 ‰).
*variable salinity*	The capability of an organism to live in environments of variable salinity (18 – 40 ‰).
*reduced salinity*	The capability of an organism to live in brackish water having a wide range of salinity between 18 ‰ and 30 ‰.
*low salinity*	The capability of an organism to live in brackish water with low salinity (< 18 ‰).
**Survival temperature**	The range of temperature in which an organism is capable to survive and grow.
Modalities:	
*cold waters*	The capability of an organism to live in cold water environments (< 0 – 10 °C).
*warm / temperate / subtropical waters*	The capability of an organism to live in environments of average temperatures (10 – 25 °C).
*tropical waters*	The capability of an organism to live in warm water environments (> 25 °C).
**Tolerance (AMBI index)**	The sensitivity of an organism to organic enrichment, classfied through the AMBI index (Borja et al. 2000).
Modalities:	
*group I*	Species very sensitive to organic enrichment and present under unpolluted conditions (initial state).
*group II*	Species indifferent to enrichment, always present in low densities with non-significant variations with time (from initial state to slightly unbalanced condition).
*group III*	Species tolerant to excess organic matter enrichment. These species may occur under normal conditions, but their populations are stimulated by organic enrichment (slightly unbalanced condition).
*group IV*	Second-order opportunistic species (slightly to pronouncedly unbalanced condition).
*group V*	First-order opportunistic species (pronouncedly unbalanced condition).
**Tube/burrow material**	Materials used for the construction of an organism’s tube or burrow (if present).
Modalities:	
*clay*	Tubes/burrows constructed of clay, a group of hydrous aluminium phyllosilicate minerals that are typically less than 3.9 micrometres in diameter.
*gravel*	Tubes/burrows constructed of gravel, an environmental material which is composed of pieces of rock that are at least two millimeters (2 mm) in its largest dimension and no more than 75 millimeters.
*sand*	Tubes/burrows constructed of sand, a naturally occurring granular material composed of finely divided rock and mineral particles.
*mud*	Tubes/burrows constructed of mud, a liquid or semi-liquid mixture of water and fine particles of silt and/or clay < 0.063 mm; the silt/clay fraction is > 80% (MarLIN 2013).
*biogenic detritus*	Tubes/burrows constructed of dead skeleton materials found in the environment (e.g. shells, algal parts).
*secretions*	Tubes/burrows constructed of bodily secretions, usually mucus.
*calcium carbonate*	Tubes constructed of calcium carbonate.
**Typically feeds on**	The type of food an organism prefers. The modalities of this trait might be expanded in the future.
Modalities:	
*algae*	Algae as food source.
*bacteria*	Bacteria as food source.
*annelids*	Annelida as food source
*ciliates*	Ciliates as food source.
*crustaceans*	Crustaceans as food source.
*diatoms*	Diatoms as food source.
*flagellates*	Flagellates as food source.
*foraminiferans*	Foraminiferans as food source.
*mollusks*	Mollusks as food source.
*detritus*	Particles of organic material from dead and decomposing organisms as food source.
*sediment*	Unselective ingestion of sediment.
*fish*	Fish, incl. their larvae, as food source.
*ascidians*	Ascidians, incl. their larvae, as food source.
*echinoderms*	Echinoderms, incl. their larvae, as food source.
*cnidarians*	Cnidarians as food source.
***Larval stage traits***	
**Habitat type of settlement / early development**	Habitat type of the larval settlement and early development after metamorphosis.
Modalities:	
*algae*	Macroalgae surfaces, such as *Laminaria* spp., or fucoids.
*biogenic reef*	Solid, massive structure which is created by accumulations of organisms, usually rising from the seabed, or at least clearly forming a substantial, discrete community or habitat which is very different from the surrounding seabed. The structure of the reef may be composed almost entirely of the reef building organism and its tubes or shells, or it may to some degree be composed of sediments, stones and shells bound together by the organisms (Holt et al. 1998).
*caves*	A hollow normally eroded in a cliff, with the penetration being greater than the width at the entrance (Sunamura 1992). Caves can also be formed by boulders (MarLIN 2013).
*crevices / fissures*	Crevices are narrow cracks in a hard substratum < 10 mm wide at its entrance, with the penetration being greater than the width at the entrance. Fissures are cracks in a hard substratum > 10 mm wide at its entrance, with the depth being greater than the width at the entrance (MarLIN 2013).
*maerl / coralligenous habitats*	A coralligenous habitat is defined by the presence of a bioherm of coralline algae grown at low irradiance levels and in relatively calm waters (Ballesteros 2006). Maerl denotes loose-lying, normally non-geniculate (i.e. not jointed), coralline red algae. Depending on the terminology used, maerl refers either to a class of rhodoliths, or may be considered distinct from rhodoliths in lacking a non-algal core. Maerl beds are composed of living or dead unattached corallines forming accumulations with or without terrigenous material (Birkett et al. 1998).
*other species*	Epibiont of other species.
*overhangs*	An overhanging part of a rock formation.
*rockpools*	A depression in the littoral zone of a rocky seashore, where, during low tide, seawater is left behind (MarLIN 2013).
*salt marsh*	A marsh whose water contains a considerable quantity of dissolved salts.
*seagrass*	Habitat associated with seagrass meadows communities. Seagrasses are flowering plants that are adapted to living fully submerged and rooted in estuarine and marine environments (MarLIN 2013).
*strandline*	A line on the shore composing debris deposited by a receding tide; commonly used to denote the line of debris at the level of Extreme High Water (MarLIN 2013).
*under boulders*	Under unattached rocks that can be very large (> 1024 mm), large (512 – 1024 mm) or small (256 – 512 mm) (MarLIN 2013).
*water column*	Pelagic habitat.
*soft sediments*	Deposits with a high water content (near or above the liquid limit), where the percolating skeleton is made of fine-grained soils (clay fraction above ~ 20%), with a high degree of saturation, and subjected to low effective confinement (Klein and Santamarina 2005).
**Juvenile mobility**	The capability of a juvenile to move spontaneously and freely.
Modalities:		
*crawler*	An organism that moves along on the substratum via movements of its legs, appendages (e.g. parapodia and chaetae) or muscles (MarLIN 2013).
*burrower*	An organism that lives or moves in a burrow in soft sediments.
*swimmer*	An organism that moves through the water column via movements of its fins, legs or appendages, via undulatory movements of the body or via jet propulsion; includes pelagic phases during reproduction (swarming at the surface) (MarLIN 2013).
*non-motile / semi-motile*	Permanently attached to a substratum (non-motile) or capable of moving across (or through) it (semi-motile) (MarLIN 2013).
**Larval development**	The mode of development from the larval to the adult stage.
Modalities:		
*direct development*	There are no intermediate larval stage(s) or postembryonic metamorphoses of any kind. Embryonic development culminates in the hatching or birth of a fully formed, albeit miniature adult (Hall and Olson 2003).
*indirect development*	One or more successive, free-living larval stages intervene between embryo and adult, with a more-or-less abrupt transition, or metamorphosis, between the last larval stage and the adult (Hall and Olson 2003).
**Larval feeding type**	The existence of two distinct larval types, feeding and non-feeding, has established the current paradigm for larval ecology. Feeding larvae are those that can capture and use exogenous food, whereas non-feeding larvae are those that cannot capture or use exogenous food (McEdward 1995).
Modalities:		
*planktotrophic*	A larval development strategy in which small eggs are converted into larger juveniles by means of larval feeding and growth (Levin and Bridges 1995).
*maternally derived nutrition*	“Umbrella term” describing the maternal sources of nutrition and including the terms lecithotrophy, adelphophagy, and translocation of nutrients.
**Larval mode of development**	Larvae development in the water column or on/in soft- or hard-bottom substrates
Modalities:		
*benthic*	Development on or near the bottom of a water body.
*pelagic*	Development in the water column.
**Location of parental care**	Defines the location of the parental care (if provided), either near the body of the parent or at a distance from it.
Modalities:		
*outside microenvironment of the parent*	Parental care is provided through e.g. protective structures, but not on the body of the parent or in its immediate living environment (e.g. in a burrow, tube or nest).
*within microenvironment of the parent*	Parental care is provided either on the body of the parent or in its immediate living environment (e.g. in a burrow, tube or nest).
**Metamorphosis amount**	Generally, any anatomical remodelling between opposing life-history periods, i.e. between the larval and the adult stage, can be considered as a form of metamorphosis (Nielsen 2000, Nielsen 2009). These changes can be rapid and cataclysmic, or can proceed gradually, depending on the particular developmental basis for the juvenile body plan within the body of the larva (Bishop et al. 2006).
Modalities:		
*catastrophic*	The metamorphosis is accompanied by massive internal change coupled with catastrophic destruction of the larval tissues. Huge chunks of the larval body, its tissues and organs, are digested away and reabsorbed, or simply discarded (Ryan 2011).
*non-catastrophic*	The adult develops from the juvenile through a process of extension and differential growth, including different larval stages but without a drastic change of the body plan.
**Parental care / Brood protection**	Any parental trait that enhances the fitness of a parent’s offspring after the offspring is released from the female body (Smiseth et al. 2012). Viviparity and other forms of lecithotrophy are excluded here from this definition and not considered as forms of parental care.
Modalities:		
*yes*	“Umbrella term”. Used to capture information that a species provides parental care to its offspring.
*no*	Used to capture information that a species does not provide parental care to its offspring beyond supplying them with a small package of yolk that serves as an initial source of nutrition until the offspring are fully capable of feeding for themselves (Smiseth et al. 2012).
**Substrate type of settlement**	Settlement is defined as the behavioural performance when pelagic larvae descend from the plankton to the benthos, and move upon the substratum with or without attaching to it. Settlement is reversible: a larva can swim up again from the substrate to resettle at another location (Qian and Dahms 2006). The surface on which larvae choose to settle is defined as the substrate of settlement.
Modalities:		
*hard substrates*	“Umbrella term”. Used to capture information that larvae choose some type of hard substrate for their settlement.
*sand*	Particles defined in three size categories: very coarse sand and granules (1 – 4 mm); medium and coarse sand (0.25 – 1 mm); very fine and fine sand (0.063 – 0.25 mm) (MarLIN 2013).
*mud*	Fine particles of silt and/or clay, < 0.063 mm diameter; the silt/clay fraction is > 80% (MarLIN 2013).
*clay*	Sediment characterised by an average particle diameter between 1 and 3.9 micrometers.
*silt*	Sediment characterised by an average particle diameter between 3.9 and 63 micrometers.
*gravel*	An environmental material which is composed of pieces of rock that are at least two millimeters (2 mm) in its largest dimension and no more than 75 millimeters.
*pebbles*	Sediment characterised by an average particle diameter between 4 and 64 mm.
*cobbles*	Sediment characterised by an average particle diameter between 64 and 256 mm.
*boulders*	Sediment characterised by an average particle diameter greater than 256 mm.
*bacterial / organic biofilm*	A complex aggregation of microorganisms marked by the excretion of a protective and adhesive matrix; usually adhering to a substratum.
***Reproduction traits***	
**Age at reproductive maturity**	Beginning of the first sexual reproductive cycle. Defined as the first ripening of gametes.
Modalities:	
*≤ 2 months*	Reproductive maturity reached at an age younger than 2 months.
*2 – 6 months*	Reproductive maturity reached at an age between 2 to 6 months.
*6 months–1 year*	Reproductive maturity reached at an age between 6 months to a year.
*1 – 2 years*	Reproductive maturity reached at an age between 1 to 2 years.
*2 – 3 years*	Reproductive maturity reached at an age between 2 to 3 years.
*3 – 4 years*	Reproductive maturity reached at an age between 3 to 4 years.
*≥ 4 years*	Reproductive maturity reached at an age more than 4 years.
**Developmental mechanism**	The mechanism of the development of the embryo(s), inside or outside of the parental organism.
Modalities:	
*oviparous*	Reproduction in which eggs are released by the female; development of offspring occurs outside the mother's body.
*viviparous*	Reproduction in which fertilization and development take place within the female body and the developing embryo derives nourishment from the female.
**Egg size**	Diameter of the eggs spawned by an organism.
Modalities:	
*0–100 μm*	Egg diameter up to 100 μm.
*100–200 μm*	Egg diameter from 100 μm to 200 μm.
*> 200 μm*	Egg diameter larger than 200 μm.
**Epitoky**	Form of reproduction of marine polychates in which the new individual arises by modification and separation from the posterior end of the worm in order to leave the bottom and reproduce (MarLIN 2013).
Modalities:	
*yes*	The organism undergoes epitokous metamorphosis.
*no*	The organism does not undergo epitokous metamorphosis.
**Factors triggering reproduction**	Factors that can initiate or enhance reproduction.
Modalities:	
*lunar cycle*	Reproduction which is timed to particular phases of the lunar cycle (or the semilunar cycle of spring and neap tides) (Dorresteijn and Westheide 1999).
*pheromones / hormones*	Spawning as a result of a pheromonal interaction between swarming males and females. Hormonal factors may be involved not only in the timing of reproduction but also in sexual differentiation (Dorresteijn and Westheide 1999).
*photoperiod*	Reproduction which is timed to a particular daylight length (Dorresteijn and Westheide 1999).
*temperature*	Reproduction which is controlled by changes in water temperature. In some species, a certain temperature value is a prerequisite for reproduction to occur (Dorresteijn and Westheide 1999).
*salinity*	Reproduction which is stimulated by changes in salinity (George 1966).
**Fecundity**	The potential reproductive capacity of an organism or population, measured by the number of gametes (eggs) or asexual propagules (MarLIN 2013).
Modalities:	
*1 – 50*	Up to 50 eggs per female and reproductive event.
*50 – 500*	From 50 to 500 eggs per female and reproductive event.
*500 – 2500*	From 500 to 2500 eggs per female and reproductive event.
*2500 – 10000*	From 2500 to 10000 eggs per female and reproductive event.
*10000 – 20000*	From 10000 to 20000 eggs per female and reproductive event.
*20000 – 100000*	From 20000 to 100000 eggs per female and reproductive event.
*> 100000*	More than 100000 eggs per female and reproductive event.
**Fertilization**	Location of the union of male and female gametes.
Modalities:	
*internal*	Fertilization takes place within the female's body.
*external (broadcast spawner)*	A method of reproduction during which the gametes (egg and sperm) unite outside the body.
*external (pseudocopulation)*	A form of external fertilization in which the partners are in close contact (Rouse and Pleijel 2006).
**Mode of reproduction**	The production by an organism of new individuals that contain some portion of genetic material inherited from that organism.
Modalities:	
*gonochoristic*	Having separate sexes throughout life (MarLIN 2013).
*simultaneous hermaphrodite*	Condition of hermaphroditic animals (and plants) in which the reproductive organs of both sexes are present and functional at the same time.
*sequential hermaphrodite*	Sequential hermaphrodites are born as one sex, but can later change into the opposite sex. Can be subdivided into protandrous and protogynous hermaphroditism.
*asexual reproduction*	Reproduction that is not sexual; that is, reproduction that does not include recombining the genotypes of two parents. Includes all different types of asexual reproduction (budding; parthenogenesis etc).
**Pattern of oogenesis**	Process of germ cell development in the female from the primordial germ cells through oogonia to the mature haploid ova. In polychaetes, two patterns have been identified: intraovarian and extraovarian (Eckelbarger 1983).
Modalities:	
*intraovarian*	Occurs when oocytes are retained by the ovary until most or all of oogenesis (and vitellogenesis) is completed. Ovaries are usually large, structurally complex, and persistent throughout the sexual phase of the female (Rouse and Pleijel 2006).
*extraovarian*	Occurs when small, previtellogenic oocytes are released from the ovary and complete vitellogenesis in the fluid-filled coelom. Ovaries are generally small, simple and sometimes have a transient nature (Rouse and Pleijel 2006).
**Population sex ratio**	The ratio of male to female (or vice versa) in a population.
Modalities:	
*1:1*	The ratio of female to male in the population is 1 to 1.
*female > male*	The number of females is higher in a population.
*female < male*	The number of males is higher in a population.
**Reproduction strategy of the individual**	Number of times an individual can reproduce during its lifetime.
Modalities:	
*iteroparous*	Breeding several times per lifetime.
*semelparous*	Organisms that have only one brood during their life time and then the parent usually dies.
**Reproduction temperature**	Water temperature that initiates or enhances reproduction.
Modalities:	
*cold water*	Reproduction in cold water environments (< 0 – 10 °C).
*warm / temperate / subtropical waters*	Reproduction in environments of average temperatures (10 – 25 °C).
*tropical waters*	Reproduction in warm water environments (> 25 °C).
**Resorption of eggs**	Ability to resorb all or part of the gametes instead of spawning them normally.
Modalities:	
*yes*	Organisms that have the ability of egg resorption.
*no*	Organisms that do not have the ability of egg resorption.
**Sexual metamorphosis**	Conspicuous change in the organism's body structure prior to reproduction.
Modalities:	
*yes*	Organisms that undergo sexual metamorphosis.
*no*	Organisms that do not undergo sexual metamorphosis.
**Spawning frequency of the population**	Period and frequency of spawning in a population.
Modalities:	
*continuous or semi-continuous*	Reproduction occurs all year round or for the most part of the year.
*annually; seasonal*	Yearly over a drawn out period of several weeks or a few months, or always in a defined season, peaks or epidemic swarming can occur within this period.
*multiple events/year*	More than once per year, but in relatively defined peaks or intense periods that do not fall within a drawn-out period.
**Sperm type**	Different types of sperm that occur in organisms and fertilize the eggs. Rouse and Jamieson (1987) proposed a system of classifying polychaete sperm based purely on function, using the terminology ect-aquasperm, ent-aquasperm and introsperm.
Modalities:	
*ect - aquasperm*	Type of sperm that are released into the water and fertilize similarly released eggs (Rouse 2005).
*ent - aquasperm*	Type of sperm that are released freely into the ambient water but differ from ect-aquasperm in being gathered by, or in some other way reaching, the female (Rouse 2005).
*introsperm*	Have no contact with water when passed from male to female (Rouse 2005).
**Synchronization of spawning**	Level of synchronization of the reproductive activity in a population.
Modalities:	
*yes*	Organisms whose populations undergo through a synchronized ripening of the gametes, usually culminating in an epidemic spawning event.
*no*	Organisms whose populations do not undergo through a synchronized ripening of the gametes.

**Table 4.** List of fields returned by the *Polytraits* database, either when downloading information from the *Polytraits* download page (export format "default CSV file") or when accessing the database through the REST API.

Column label	Column description
Taxon	The taxon for which the information was recorded.
Author	The author and year of the *Taxon* for which the information was recorded.
Valid taxon	Currently accepted name of the *Taxon* (as stored in the *Polytraits* database – information might not be up to date with the latest taxonomic literature in some cases). If *Taxon* is currently accepted, this field contains the same value as *Taxon*).
Valid author	Currently accepted name of the *Author* (as stored in the *Polytraits* database – information might not be up to date with the latest taxonomic literature in some cases). If *Taxon* is currently accepted, this field contains the same value as *Author*.
Taxonomic status	Information on why *Taxon* is not currently valid (e.g. objective synonym). If *Taxon* and *Author* are currently accepted, this field is empty.
Source of synonymy	Literature reference for the *Taxonomic status* (if present).
Parent taxon	The *Taxon*'s direct parent in the taxonomic classification (as stored in the *Polytraits* database).
Trait	The biological trait for which information is available (e.g. "Feeding type").
Modality	The sub-category of the *Trait* for which information is available (e.g. "Carnivore").
Modality abbreviation	An abbreviated version of the often verbose *Modality* – useful as a label in further analyses of the data (e.g. "FEED_C").
Traitvalue	Describes the affinity of the *Taxon* to the above *Modality* – can be either "0" (for "absent") or "1" (for "present").
Reference	Literature reference leading to the assignment of the *Traitvalue* to the *Modality* for the *Taxon*.
DOI	Digital Object Identifier (where available) of the *Reference*.
Value creator	Person who assigned the *Traitvalue* to the *Modality* for the *Taxon*, supported by a *Reference*.
Value creation date	Date and time when the above information was entered into the database.
Text Excerpt	A quotation of the original text passage from the literature source that led to the assignment of assignment of the *Modality* / *Traitvalue* to the *Taxon*. Empty if information has not been recorded yet.
Text Excerpt creator	Person who entered the *Text Excerpt*. Only present if *Text Excerpt* is present.
Text Excerpt creation date	Date and time when the *Text Excerpt* was entered into the database. Only present if *Text Excerpt* is present.

**Table 5.** A method to search for taxa in the *Polytraits* database. Returns matching taxon identifiers. URL: http://polytraits.lifewatchgreece.eu/taxon/{query}/{format}/?{*other parameter key value pairs*} Example: Retrieve all taxon information about taxa starting with "*Syllis*" and return them as a numeric array: http://polytraits.lifewatchgreece.eu/taxon/Syllis/json/?exact=0&verbose=1&assoc=0

****Parameter name****	****data type****	**allowed values**	**default value**	****Parameter Note****	**Output**
query	string	any taxon name		spaces have to be url-encoded, case-insensitive	
format	string	json|xml	json	Optional. Currently only json is implemented	
exact	boolean	1|0	1	optional	When true, returns only the exact match for the query string. When false, returns all matches beginning with the query string.
verbose	boolean	1|0	0	optional	When true, returns per taxon: *array (taxon ID, taxon name, authority, valid taxon ID, valid taxon name, valid authority, status, source of synonymy, rank)*. When false, returns per taxon: *array (taxon ID, taxon name*).
assoc	boolean	1|0	0	optional	When true, an associative array is returned with the taxon ID as keys. When false, a numeric array is returned.

**Table 6.** A method to retrieve trait information for one or more taxon identifiers from the *Polytraits* database. Returns all trait information for the given IDs. URL: http://polytraits.lifewatchgreece.eu/traits/{query}/{format}/?{*other parameter key value pairs*} Example: Retrieve all trait information about the taxa with ID 1 and 2 and return them as an associative array with the taxon IDs as keys: *http://polytraits.lifewatchgreece.eu/traits/1,2/json/?verbose=1&assoc=1* The output fields are documented in Table 4.

****Parameter name****	****data type****	**allowed values**	**default value**	****Parameter Note****	**Output**
query	integer or string	one or more taxon identifiers, comma-separated		Use “taxon” method to retrieve a list of IDs. Up to 10 Ids are allowed	
format	string	json|xml	json	Optional. Currently only json is implemented	
verbose	boolean	1|0	1	Optional	When true, returns per taxon the fields documented in Table 4. When false, returns per taxon: *array (trait, modality, traitvalue).*
assoc	boolean	1|0	1	optional	When true, an associative array is returned with the taxon ID as keys. When false, a numeric array is returned.
