# Supplementary material for: Polytraits: A database on biological traits of marine polychaetes
Source: Biodivers Data J. 2014 Jan 17;(2):e1024. doi: 10.3897/BDJ.2.e1024 (PMC4030217; doi:10.3897/BDJ.2.e1024)
Supplement: Supplementary material 1 — Database documentation [file biodiversity_data_journal-2-e1024-s001.pdf]

Database documentation

Author *Sarah Faulwetter*

Date*: 05 November 2013*

Description of the database tables contained in the regular database dumps that are made availabel for download on the website. The dump only contains tables that contain relevant biological traits data - "helper" tables are not included. Likewise, some fields in the download are only used for certain functions of the web interface, these are described below as "internally used only" and greyed out.

Note: the field "creator" in each table links to the database user table which is not exported for security reasons. For proper citation of the records (if needed) please consult the list of contributors on the website.

Tables

taxa	*Taxonomic backbone of the database, contains all taxonomic names their rank and their synonymy. The hierarchy is stored both as a parent-child model as well as a nested set model.*
**field name**	**description**
id_taxon	primary key of the table
taxon	the taxon name without authority
author	the authority and year of the taxon's description
fk_valid	*foreign key*, links to id_taxon in table *taxa* and points to the name/taxon that is currently considered as the accepted one. In case of no synonymy, fk_valid and id_taxon have the same value
fk_unacc_reason	*foreign key,* links to table *taxa_unacc_reason*, describes the reason for synonymy (e.g. objective synonym, subjective synonym, ...)
fk_parent	*foreign key* links to id_taxon in table *taxa* and points to the direct parent of the current taxon in the taxonomic classification
lft	left value of a nested set hierarchy model
rgt	right value of a nested set hierarchy model
fk_rank	*foreign key*, links to table *ranks*
fk_publication_synonym	*foreign key*, links to table *publications* and points to the publication which contains information on the synonymy of the taxon
datemodify	date of last modification of this entry
creator	user who created this entry

ranks	*contains a list of the biological ranks (e.g. species, genus...) and their hierarchy*
**field name**	**description**
id_rank	primary key of the table
rank	name of the rank (e.g. species, genus)
ranklevel	*internally used only*
fk_parentrank	*foreign key*, links to id_rank in table *rank*,stores the next higher rank in the hierarchy
parent_rankgroup	*internally used only*
rankpath	*internally used only*
datemodify	date of last modification of this entry
creator	user who created this entry

taxa_unacc_ reason	*stores a list of reasons why a taxonomic name is not accepted (e.g. synonymy, misspelling)*
**field name**	**description**
id_unacc_reason	primary key of the table
unaccept_reason	list of reasons for synonymy (e.g. objective synonym, subjective synonym, ...)

modalities	*stores all modalities (=trait categories) used in the database*
**field name**	**description**
id_modality	primary key of the table
modality	name of the modality
modality_shortname	abbreviated name of the modality
fk_trait	***foreign key,*** links to table ***traits***
rule_present	*internally used only*
definition	definition of the modality. Can contain literature references in the form “[123]” which correspond to *id_publication* in table *publications.*
term_identifier	unique identifier of the term in form of a URL, pointing to the term's definition
related_terms	related, similar or synonymous terms which are often found in the literature
description	any additional explanations to clarify the scope of the term. Can contain literature references in the form “[123]” which correspond to *id_publication* in table *publications.*
sorting_weight	*internally used only*
temp_trait	*internally used only*
temp_reference	*internally used only*
datemodify	date of last modification of this entry
creator	user who created this entry

traits	*stores all traits used in the database*
**field name**	**description**
id_trait	primary key of the table
trait	name of the trait
fk_mode	foreign key, links to table *modes*
trait_shortname	abbreviated name of the trait
definition	definition of the trait. Can contain literature references in the form “[123]” which correspond to *id_publication* in table *publications.*
related terms	related, similar or synonymous terms which are often found in the literature
term_identifier	unique identifier of the term in form of a URL, pointing to the term's definition
description	any additional explanations to clarify the scope of the term. Can contain literature references in the form “[123]” which correspond to *id_publication* in table *publications.*
datemodify	date of last modification of this entry
creator	user who created this entry

modes	*a list that groups the traits into adult traits, reproductive traits and larval traits*
**field name**	**description**
id_mode	primary key of the table
mode	name of the mode
datemodify	date of last modification of this entry
creator	user who created this entry

publications	*stores all publications used in the database*
**field name**	**description**
id_publication	primary key of the table
pub_year	the journal's title
title	title of the publication
volume	volume of the publication (where applicable, e.g. journals)
pages	page range or number of pages of the publication
issue	issue of the publication (where applicable, e.g. journals)
other	any description of a publication not classifiable into a publication type such as journal article, book, etc.
label	*internally used only*
DOI	Digital Object identifier of the publication
fk_journal	foreign key, links to table *journals*
booktitle	title of the publication if it is a monograph-type of publication (e.g. book, report)

publisher	publisher of the publication
city	city of the publisher
institution	name of the institution that published the publication (mainly applied to theses or reports)
seriestitle	title of the book or report series
thesistype	if publication is a thesis, this stores the type of thesis (e.g. BSc, MSc, PhD)
referencetype	type of the publication (e.g. journal article, book, book chapter, thesis, report, URL...)
abstract	abstract of the publication
url	Unified Resource Locator - internet address linking to the publication
fullref	automatically assembled full citation of the reference
shortref	short version of the reference (e.g. Smith et al. 1999)
datemodify	date of last modification of this entry
creator	user who created this entry

keywords	*a list of keywords with which publications can be annotated*
**field name**	**description**
id_keyword	primary key of the table
keyword	the term / keyword
datemodify	date of last modification of this entry
creator	user who created this entry

keyword_pub	*links table “publication” to table “keywords”*
**field name**	**description**
fk_keyword	foreign key, linking to table *keywords*
fk_pub	foreign key, linking to table *publications*
datemodify	date of last modification of this entry
creator	user who created this entry

persons	*a list of all authors and editors of publications stored in the database*
**field name**	**description**
id_person	primary key of the table
last_name	last name of the author/ editor
first_name	first name of the author/ editor
datemodify	date of last modification of this entry
creator	user who created this entry

pers_pub	*links table "publications" to table "persons"*
**field name**	**description**
fk_pers	foreign key, linking to table *persons*
fk_pub	foreign key, linking to table *publications*
order_appear	stores the order of the authors in a given publication
flag_editor	flags a person to be an editor of the given publication
datemodify	date of last modification of this entry
creator	user who created this entry

journals	*a list of all journal names of publications stored in the database*
**field name**	**description**
id_journal	primary key of the table
journal	the journal's title
datemodify	date of last modification of this entry
creator	user who created this entry

sourcedata	*stores the exact text passage that lead to the assignment of a modality to a taxon*
**field name**	**description**
id_sourcedata	primary key of the table
sourcedata	contains the exact text passage supporting the assignment of a modality to a taxon
date_modify	date of last modification of this entry
creator	user who created this entry

relations	*Links tables "taxon" and "modalities" and assigns a present/absent value to this relationship, indicating whether a modality is present or absent in a taxon*
**field name**	**description**
id_relation	primary key of the table
fk_taxon	foreign key, links to table *taxa*
fk_modality	foreign key, links to table *modalities*
traitvalue	present/absent value, describes whether a modality is present in a taxon or not
datemodify	date of last modification of this entry
creator	user who created this entry

relation_pub	*links table "relations" to table "publications" to support each taxon-modality-traitvalue entity to a publication*
**field name**	**description**
fk_relation	foreign key, links to table *relations;* primary key in combination with fk_publication
fk_publication	foreign key, links to table *publications*; primary key in combination with fk_relation
fk_original	foreign key, links to table *sourcedata*
datemodify	date of last modification of this entry
creator	user who created this entry

users	*Lists all users of the database*
**field name**	**description**
user	foreign key, links to field "creator" in all other tables
firstname	First name of the user
lastname	Last name of the user

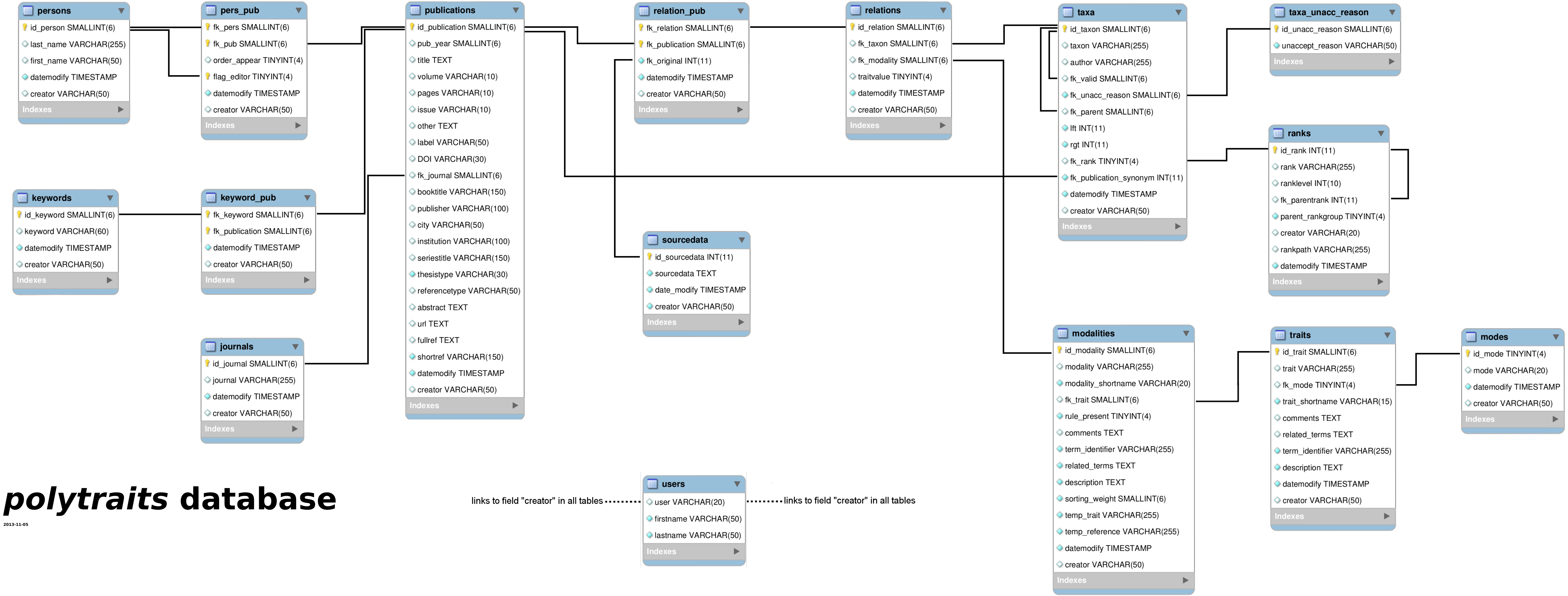

polytraits **database**

2013-11-05
